# 2,4-Bis(furan-2-yl)-1,5-dimethyl-3-aza­bicyclo­[3.3.1]nonan-9-one

**DOI:** 10.1107/S1600536813010180

**Published:** 2013-04-20

**Authors:** R. Venkateswaramoorthi, S. Rizwana Begum, R. Hema, K. Krishnasamy, A.G. Anitha

**Affiliations:** a Department of Chemistry, Annamalai University, Annamalai Nagar 608 002, India; b Department of Physics, Seethalakshmi Ramaswami College (Autonomous), Tiruchirappalli 620 002, India

## Abstract

In the title compound, C_18_H_21_NO_3_, the bicyclic ring system adopts a twin-chair conformation. The two methyl groups attached to the bicycle are in an equatorial orientation for both rings. One of the furan rings is disordered over two orientations with an occupancy ratio of 0.686 (6):0.314 (6). In the crystal, very long N—H⋯O hydrogen bonds connect the mol­ecules into a chain perpendicular to the *ac* plane.

## Related literature

For the synthesis and biological activity of 3-aza­bicyclo­[3.3.1] nonan-9-ones, see: Parthiban *et al.* (2009 ▶); Hardick *et al.* (1996 ▶); Jeyaraman & Avila (1981 ▶). For puckering parameters, see: Cremer & Pople (1975 ▶).
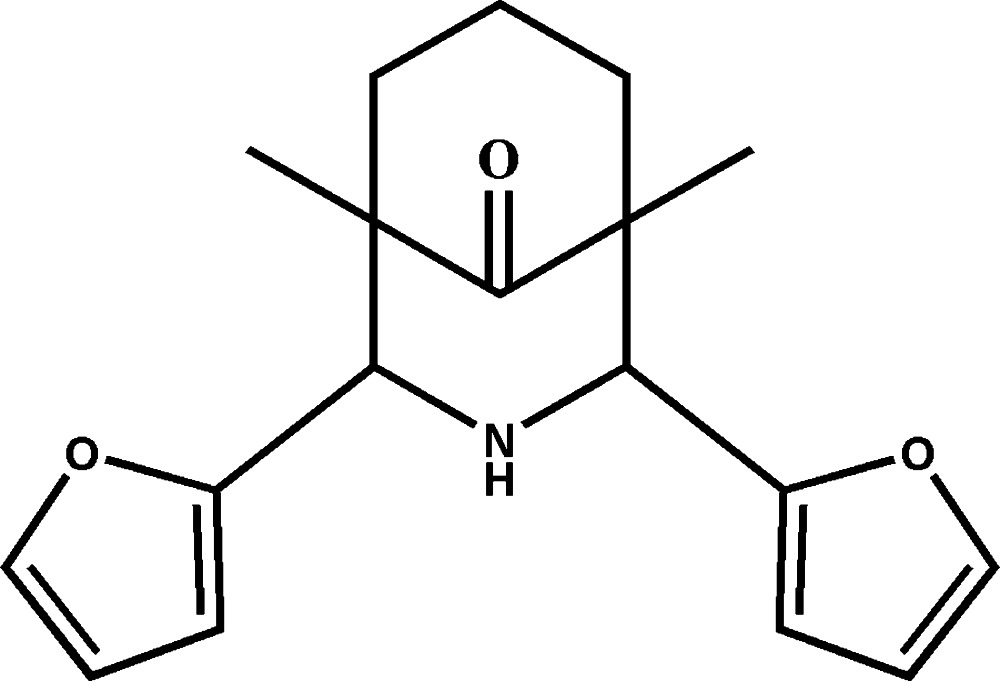


## Experimental

### 

#### Crystal data

C_18_H_21_NO_3_
*M* *_r_* = 299.36
Monoclinic, 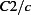

*a* = 21.268 (2) Å
*b* = 6.7031 (9) Å
*c* = 22.303 (2) Å
β = 92.776 (6)°
*V* = 3175.7 (6) Å^3^
*Z* = 8
Mo *K*α radiation
μ = 0.09 mm^−1^
*T* = 293 K
0.30 × 0.25 × 0.20 mm

#### Data collection

Bruker SMART APEX CCD area-detector diffractometer
11487 measured reflections
2784 independent reflections
2127 reflections with *I* > 2σ(*I*)
*R*_int_ = 0.031

#### Refinement

*R*[*F* ^2^ > 2σ(*F* ^2^)] = 0.044
*wR*(*F* ^2^) = 0.130
*S* = 1.05
2784 reflections
252 parameters
220 restraints
H atoms treated by a mixture of independent and constrained refinement
Δρ_max_ = 0.19 e Å^−3^
Δρ_min_ = −0.20 e Å^−3^

### 

Data collection: *APEX2* (Bruker, 2008 ▶); cell refinement: *SAINT* (Bruker, 2008 ▶); data reduction: *SAINT*; program(s) used to solve structure: *SHELXS97* (Sheldrick, 2008 ▶); program(s) used to refine structure: *SHELXL97* (Sheldrick, 2008 ▶); molecular graphics: *ORTEP-3 for Windows* (Farrugia, 2012 ▶); software used to prepare material for publication: *SHELXL97* and *PLATON* (Spek, 2009 ▶).

## Supplementary Material

Crystal structure: contains datablock(s) I, global. DOI: 10.1107/S1600536813010180/aa2083sup1.cif

Structure factors: contains datablock(s) I. DOI: 10.1107/S1600536813010180/aa2083Isup2.hkl

Supplementary material file. DOI: 10.1107/S1600536813010180/aa2083Isup3.cml

Additional supplementary materials:  crystallographic information; 3D view; checkCIF report

## Figures and Tables

**Table 1:** Hydrogen-bond geometry (Å, °)

*D*—H⋯*A*	*D*—H	H⋯*A*	*D*⋯*A*	*D*—H⋯*A*
N1—H1⋯O1^i^	0.89 (2)	2.84 (2)	3.647 (2)	152.0 (13)

Symmetry code: (i) 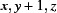.

**Fig. 1.**
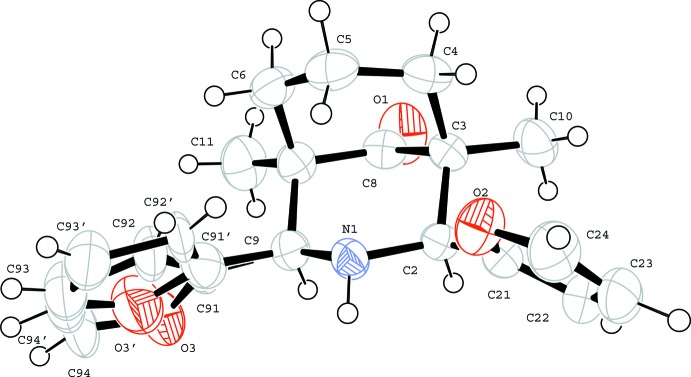
View of the molecule of the title compound showing the atom-labelling scheme. Displacement ellipsoids are drawn at the 50% probability level. H atoms are represented by circles of arbitrary radii.

## supplementary crystallographic information

### Comment

Molecules with the 3-azabicyclo[3.3.1]nonane nucleus are of great interest due to their presence in a wide variety of naturally occurring diterpenoid/norditerpenoid alkaloids and their broad-spectrum biological activities such as antimicrobial, analgesic, antogonistic, anti-inflammatory, local anesthetic hypotensive activity and so on (Parthiban *et al.*, 2009; Hardick *et al.*, 1996; Jeyaraman & Avila, 1981). Hence, the synthesis of new molecules with the 3-azabicyclo[3.3.1] nonane nucleus and their stereochemical investigations are of interest in the field of medicinal chemistry. Also, the stereochemistry of the synthesized molecules is a major criterium for their biological response. Hence, it is important to establish the stereochemistry of the bio-active molecules. As a consequence, the present study was undertaken to examine the configuration and conformation of the synthesized title compound.

In the crystal structure of the title compound, the bicycle system adopts twin-chair conformation with puckering parameters Q = 0.571 (2) Å, θ = 178.3 (2)° and φ = 360 (5)° for the piperidine ring N1/C2/C3/C8/C7/C9 and Q = 0.568 (2) Å, θ = 168.0 (2)° and φ = 121.4 (10)° for the cyclohexanone ring C3/C4/C5/C6/C7/C8 (Cremer & Pople, 1975).

The dihedral angle between the furyl ring C21/C22/C23/C24/O2 and the piperidine ring N1/C2/C3/C8/C7/C9 is 70.09 (8)° and the disordered furyl ring makes with the same piperidine ring two dihedral angles of 70.83 (23)° for major part C91/C92/C93/C94/O3 and 67.40 (36)° for minor part C91/C92'/C93'/C94'/O3'. The methyl groups attached at C7 and C3 are in equitorial orientation with torsion angles of 175.31 (19)° (N1–C9–C7–C11) and -174.72 (17)° (N1–C2–C3–C10).

### Experimental

The title compound was obtained by condensation of 2,6-dimethylcyclohexanone, furfuraldehyde and ammonium acetate in 1:2:1 ratio in ethanol. The reaction mixture was warmed and stirred until the completion of reaction. The crude product was washed with an ethanol - ethyl ether (1:5) mixture and recrystallized from ethanol - chloroform (1:1) to obtain the pure compound.

### Refinement

The methyl H atoms were constrained to an ideal geometry (C—H = 0.96 Å) with *U*_iso_(H) = 1.5*U*_eq_(C), but were allowed to rotate freely about the C—C bonds. All remaining H atoms were placed in geometrically idealized positions (C—H = 0.93–0.98 Å) and constrained to ride on their parent atoms with *U*_iso_(H) = 1.2*U*_eq_(C). In order to bring the *U^ij^* parameters of the disordered atoms to the tolerant levels the restraints SADI, SIMU and ISOR were used.

### Crystal data

**Table:**

C_18_H_21_NO_3_	*F*(000) = 1280
*M**_r_* = 299.36	*D*_x_ = 1.252 Mg m^−^^3^
Monoclinic, *C*2/*c*	Mo *K*α radiation, λ = 0.71073 Å
Hall symbol: -C 2yc	Cell parameters from 11487 reflections
*a* = 21.268 (2) Å	θ = 1.8–28.4°
*b* = 6.7031 (9) Å	µ = 0.09 mm^−^^1^
*c* = 22.303 (2) Å	*T* = 293 K
β = 92.776 (6)°	Block, brown
*V* = 3175.7 (6) Å^3^	0.30 × 0.25 × 0.20 mm
*Z* = 8	

### Data collection

**Table:**

Bruker SMART APEX CCD area-detector diffractometer	2127 reflections with *I* > 2σ(*I*)
Radiation source: fine-focus sealed tube	*R*_int_ = 0.031
Graphite monochromator	θ_max_ = 25.0°, θ_min_ = 1.9°
ω scan	*h* = −23→25
11487 measured reflections	*k* = −5→7
2784 independent reflections	*l* = −22→26

### Refinement

**Table:**

Refinement on *F*^2^	Secondary atom site location: difference Fourier map
Least-squares matrix: full	Hydrogen site location: inferred from neighbouring sites
*R*[*F*^2^ > 2σ(*F*^2^)] = 0.044	H atoms treated by a mixture of independent and constrained refinement
*wR*(*F*^2^) = 0.130	*w* = 1/[σ^2^(*F*_o_^2^) + (0.0637*P*)^2^ + 1.7182*P*] where *P* = (*F*_o_^2^ + 2*F*_c_^2^)/3
*S* = 1.05	(Δ/σ)_max_ < 0.001
2784 reflections	Δρ_max_ = 0.19 e Å^−^^3^
252 parameters	Δρ_min_ = −0.20 e Å^−^^3^
220 restraints	Extinction correction: *SHELXL97* (Sheldrick, 2008), Fc^*^=kFc[1+0.001xFc^2^λ^3^/sin(2θ)]^-1/4^
Primary atom site location: structure-invariant direct methods	Extinction coefficient: 0.0024 (5)

### Special details

**Table:**

Geometry. All e.s.d.'s (except the e.s.d. in the dihedral angle between two l.s. planes) are estimated using the full covariance matrix. The cell e.s.d.'s are taken into account individually in the estimation of e.s.d.'s in distances, angles and torsion angles; correlations between e.s.d.'s in cell parameters are only used when they are defined by crystal symmetry. An approximate (isotropic) treatment of cell e.s.d.'s is used for estimating e.s.d.'s involving l.s. planes.
Refinement. Refinement of *F*^2^ against ALL reflections. The weighted *R*-factor *wR* and goodness of fit *S* are based on *F*^2^, conventional *R*-factors *R* are based on *F*, with *F* set to zero for negative *F*^2^. The threshold expression of *F*^2^ > σ(*F*^2^) is used only for calculating *R*-factors(gt) *etc*. and is not relevant to the choice of reflections for refinement. *R*-factors based on *F*^2^ are statistically about twice as large as those based on *F*, and *R*- factors based on ALL data will be even larger.

### Fractional atomic coordinates and isotropic or equivalent isotropic displacement parameters (Å^2^)

**Table:**

	*x*	*y*	*z*	*U*_iso_*/*U*_eq_	Occ. (<1)
N1	0.17290 (7)	0.0845 (3)	0.13064 (7)	0.0446 (4)	
H1	0.1765 (10)	0.215 (4)	0.1368 (9)	0.058 (6)*	
C2	0.23315 (8)	−0.0076 (3)	0.15048 (8)	0.0391 (4)	
H2	0.2398	0.0198	0.1935	0.047*	
C3	0.23109 (8)	−0.2391 (3)	0.14260 (8)	0.0403 (4)	
C4	0.21984 (10)	−0.3083 (3)	0.07672 (8)	0.0506 (5)	
H4A	0.2530	−0.2539	0.0532	0.061*	
H4B	0.2233	−0.4526	0.0755	0.061*	
C5	0.15671 (10)	−0.2485 (3)	0.04728 (9)	0.0562 (6)	
H5A	0.1501	−0.3209	0.0098	0.067*	
H5B	0.1573	−0.1072	0.0380	0.067*	
C6	0.10205 (10)	−0.2917 (3)	0.08770 (9)	0.0544 (5)	
H6A	0.0942	−0.4342	0.0873	0.065*	
H6B	0.0646	−0.2267	0.0706	0.065*	
C7	0.11233 (9)	−0.2234 (3)	0.15358 (8)	0.0455 (5)	
C8	0.17479 (9)	−0.3139 (3)	0.17506 (8)	0.0444 (5)	
C9	0.11855 (8)	0.0068 (3)	0.16110 (9)	0.0461 (5)	
H9	0.1259	0.0337	0.2041	0.055*	
C10	0.29224 (10)	−0.3283 (3)	0.16918 (10)	0.0595 (6)	
H10A	0.3007	−0.2761	0.2088	0.089*	
H10B	0.3262	−0.2941	0.1443	0.089*	
H10C	0.2883	−0.4708	0.1711	0.089*	
C11	0.05852 (10)	−0.2986 (4)	0.19039 (11)	0.0701 (7)	
H11A	0.0591	−0.4417	0.1914	0.105*	
H11B	0.0191	−0.2537	0.1724	0.105*	
H11C	0.0635	−0.2476	0.2306	0.105*	
C21	0.28552 (8)	0.0899 (3)	0.11924 (8)	0.0418 (4)	
C22	0.34330 (9)	0.1509 (3)	0.13830 (10)	0.0559 (5)	
H22	0.3611	0.1405	0.1771	0.067*	
C23	0.37202 (10)	0.2347 (3)	0.08755 (11)	0.0627 (6)	
H23	0.4122	0.2893	0.0869	0.075*	
C24	0.33033 (10)	0.2198 (3)	0.04147 (11)	0.0616 (6)	
H24	0.3368	0.2637	0.0027	0.074*	
O1	0.17859 (7)	−0.4444 (3)	0.21255 (7)	0.0744 (5)	
O2	0.27645 (6)	0.1308 (2)	0.05925 (6)	0.0564 (4)	
C91	0.0595 (4)	0.114 (2)	0.1410 (2)	0.0532 (11)	0.686 (6)
C92	0.0368 (2)	0.1727 (7)	0.0867 (2)	0.0656 (11)	0.686 (6)
H92	0.0561	0.1616	0.0503	0.079*	0.686 (6)
C93	−0.0247 (4)	0.2576 (19)	0.0973 (3)	0.0883 (17)	0.686 (6)
H93	−0.0535	0.3089	0.0686	0.106*	0.686 (6)
C94	−0.03169 (18)	0.2467 (7)	0.1581 (3)	0.0947 (15)	0.686 (6)
H94	−0.0667	0.2934	0.1772	0.114*	0.686 (6)
O3	0.02057 (15)	0.1562 (6)	0.18791 (19)	0.0811 (11)	0.686 (6)
C91'	0.0638 (9)	0.116 (5)	0.1303 (6)	0.064 (2)	0.314 (6)
C92'	0.0621 (4)	0.1568 (14)	0.0708 (4)	0.0480 (18)	0.314 (6)
H92'	0.0929	0.1318	0.0436	0.058*	0.314 (6)
C93'	0.0022 (4)	0.2467 (14)	0.0597 (5)	0.082 (2)	0.314 (6)
H93'	−0.0137	0.2875	0.0221	0.098*	0.314 (6)
C94'	−0.0289 (9)	0.265 (4)	0.1116 (7)	0.082 (2)	0.314 (6)
H94'	−0.0680	0.3245	0.1158	0.099*	0.314 (6)
O3'	0.0093 (4)	0.1787 (15)	0.1571 (5)	0.094 (2)	0.314 (6)

### Atomic displacement parameters (Å^2^)

**Table:**

	*U*^11^	*U*^22^	*U*^33^	*U*^12^	*U*^13^	*U*^23^
N1	0.0369 (8)	0.0346 (9)	0.0622 (10)	0.0025 (7)	0.0027 (7)	0.0001 (7)
C2	0.0388 (9)	0.0386 (10)	0.0398 (9)	0.0028 (8)	0.0001 (7)	−0.0037 (8)
C3	0.0432 (10)	0.0366 (10)	0.0414 (10)	0.0047 (8)	0.0041 (8)	0.0007 (8)
C4	0.0621 (12)	0.0438 (11)	0.0471 (11)	−0.0023 (9)	0.0137 (9)	−0.0073 (9)
C5	0.0743 (14)	0.0538 (13)	0.0401 (10)	−0.0032 (11)	−0.0026 (10)	−0.0072 (9)
C6	0.0555 (12)	0.0462 (12)	0.0607 (13)	−0.0067 (9)	−0.0071 (10)	−0.0054 (9)
C7	0.0431 (10)	0.0451 (11)	0.0486 (11)	−0.0034 (8)	0.0069 (8)	0.0035 (8)
C8	0.0551 (11)	0.0418 (11)	0.0365 (9)	0.0000 (8)	0.0039 (8)	0.0026 (8)
C9	0.0386 (10)	0.0501 (12)	0.0499 (11)	−0.0001 (9)	0.0054 (8)	−0.0064 (9)
C10	0.0556 (12)	0.0497 (12)	0.0733 (14)	0.0126 (10)	0.0026 (10)	0.0093 (11)
C11	0.0558 (13)	0.0720 (16)	0.0838 (16)	−0.0081 (11)	0.0176 (12)	0.0169 (13)
C21	0.0400 (10)	0.0379 (10)	0.0473 (10)	0.0038 (8)	0.0005 (8)	−0.0019 (8)
C22	0.0410 (11)	0.0607 (14)	0.0654 (13)	−0.0006 (9)	−0.0031 (9)	−0.0045 (11)
C23	0.0416 (11)	0.0576 (14)	0.0899 (17)	−0.0072 (10)	0.0141 (11)	0.0002 (12)
C24	0.0550 (13)	0.0567 (14)	0.0746 (15)	−0.0069 (10)	0.0171 (11)	0.0122 (11)
O1	0.0700 (10)	0.0808 (12)	0.0729 (10)	0.0022 (8)	0.0074 (8)	0.0381 (9)
O2	0.0516 (8)	0.0614 (10)	0.0559 (9)	−0.0083 (7)	0.0004 (6)	0.0124 (7)
C91	0.029 (2)	0.045 (2)	0.086 (3)	0.0037 (18)	0.0108 (19)	−0.010 (3)
C92	0.038 (2)	0.058 (2)	0.098 (3)	0.010 (2)	−0.018 (2)	0.007 (2)
C93	0.049 (3)	0.076 (3)	0.138 (4)	0.009 (2)	−0.016 (3)	−0.005 (4)
C94	0.0333 (18)	0.089 (3)	0.162 (4)	0.0174 (18)	0.000 (2)	−0.018 (3)
O3	0.0495 (16)	0.090 (2)	0.105 (3)	0.0131 (13)	0.0101 (16)	−0.013 (2)
C91'	0.042 (4)	0.058 (4)	0.092 (4)	−0.003 (4)	0.017 (4)	−0.014 (4)
C92'	0.025 (3)	0.054 (3)	0.064 (4)	0.016 (3)	−0.003 (3)	0.004 (3)
C93'	0.057 (4)	0.073 (4)	0.114 (5)	0.004 (4)	−0.021 (4)	0.000 (4)
C94'	0.043 (4)	0.072 (4)	0.130 (5)	0.014 (4)	−0.009 (4)	−0.002 (5)
O3'	0.066 (4)	0.084 (3)	0.133 (4)	0.008 (3)	0.009 (4)	−0.007 (4)

### Geometric parameters (Å, º)

**Table:**

N1—C9	1.464 (2)	C11—H11A	0.9600
N1—C2	1.471 (2)	C11—H11B	0.9600
N1—H1	0.89 (2)	C11—H11C	0.9600
C2—C21	1.493 (2)	C21—C22	1.344 (3)
C2—C3	1.562 (3)	C21—O2	1.370 (2)
C2—H2	0.9800	C22—C23	1.427 (3)
C3—C8	1.514 (3)	C22—H22	0.9300
C3—C10	1.525 (3)	C23—C24	1.328 (3)
C3—C4	1.548 (3)	C23—H23	0.9300
C4—C5	1.520 (3)	C24—O2	1.368 (2)
C4—H4A	0.9700	C24—H24	0.9300
C4—H4B	0.9700	C91—C92	1.342 (5)
C5—C6	1.533 (3)	C91—O3	1.393 (4)
C5—H5A	0.9700	C92—C93	1.455 (7)
C5—H5B	0.9700	C92—H92	0.9300
C6—C7	1.544 (3)	C93—C94	1.373 (7)
C6—H6A	0.9700	C93—H93	0.9300
C6—H6B	0.9700	C94—O3	1.405 (5)
C7—C8	1.517 (3)	C94—H94	0.9300
C7—C11	1.526 (3)	C91'—C92'	1.353 (8)
C7—C9	1.557 (3)	C91'—O3'	1.395 (8)
C8—O1	1.210 (2)	C92'—C93'	1.421 (7)
C9—C91	1.497 (4)	C92'—H92'	0.9300
C9—C91'	1.512 (8)	C93'—C94'	1.366 (10)
C9—H9	0.9800	C93'—H93'	0.9300
C10—H10A	0.9600	C94'—O3'	1.396 (9)
C10—H10B	0.9600	C94'—H94'	0.9300
C10—H10C	0.9600		
			
C9—N1—C2	114.00 (15)	C3—C10—H10A	109.5
C9—N1—H1	110.0 (13)	C3—C10—H10B	109.5
C2—N1—H1	107.4 (14)	H10A—C10—H10B	109.5
N1—C2—C21	109.54 (15)	C3—C10—H10C	109.5
N1—C2—C3	111.35 (14)	H10A—C10—H10C	109.5
C21—C2—C3	113.59 (14)	H10B—C10—H10C	109.5
N1—C2—H2	107.4	C7—C11—H11A	109.5
C21—C2—H2	107.4	C7—C11—H11B	109.5
C3—C2—H2	107.4	H11A—C11—H11B	109.5
C8—C3—C10	111.32 (16)	C7—C11—H11C	109.5
C8—C3—C4	105.37 (15)	H11A—C11—H11C	109.5
C10—C3—C4	109.95 (16)	H11B—C11—H11C	109.5
C8—C3—C2	107.08 (14)	C22—C21—O2	109.35 (17)
C10—C3—C2	109.08 (15)	C22—C21—C2	132.61 (18)
C4—C3—C2	113.99 (15)	O2—C21—C2	118.04 (15)
C5—C4—C3	115.06 (16)	C21—C22—C23	106.79 (19)
C5—C4—H4A	108.5	C21—C22—H22	126.6
C3—C4—H4A	108.5	C23—C22—H22	126.6
C5—C4—H4B	108.5	C24—C23—C22	106.79 (18)
C3—C4—H4B	108.5	C24—C23—H23	126.6
H4A—C4—H4B	107.5	C22—C23—H23	126.6
C4—C5—C6	112.07 (17)	C23—C24—O2	110.22 (19)
C4—C5—H5A	109.2	C23—C24—H24	124.9
C6—C5—H5A	109.2	O2—C24—H24	124.9
C4—C5—H5B	109.2	C24—O2—C21	106.86 (16)
C6—C5—H5B	109.2	C92—C91—O3	114.7 (3)
H5A—C5—H5B	107.9	C92—C91—C9	132.1 (4)
C5—C6—C7	115.29 (16)	O3—C91—C9	113.2 (3)
C5—C6—H6A	108.5	C91—C92—C93	104.8 (4)
C7—C6—H6A	108.5	C91—C92—H92	127.6
C5—C6—H6B	108.5	C93—C92—H92	127.6
C7—C6—H6B	108.5	C94—C93—C92	106.3 (5)
H6A—C6—H6B	107.5	C94—C93—H93	126.9
C8—C7—C11	111.48 (16)	C92—C93—H93	126.9
C8—C7—C6	105.26 (15)	C93—C94—O3	111.7 (5)
C11—C7—C6	109.79 (17)	C93—C94—H94	124.2
C8—C7—C9	107.09 (15)	O3—C94—H94	124.2
C11—C7—C9	109.36 (17)	C91—O3—C94	102.6 (4)
C6—C7—C9	113.80 (16)	C92'—C91'—O3'	112.1 (7)
O1—C8—C3	122.80 (18)	C92'—C91'—C9	121.8 (8)
O1—C8—C7	122.38 (17)	O3'—C91'—C9	126.1 (8)
C3—C8—C7	114.66 (15)	C91'—C92'—C93'	103.8 (7)
N1—C9—C91	111.1 (4)	C91'—C92'—H92'	128.1
N1—C9—C91'	103.0 (10)	C93'—C92'—H92'	128.1
N1—C9—C7	111.54 (15)	C94'—C93'—C92'	111.0 (10)
C91—C9—C7	112.2 (6)	C94'—C93'—H93'	124.5
C91'—C9—C7	111.8 (15)	C92'—C93'—H93'	124.5
N1—C9—H9	107.2	C93'—C94'—O3'	106.8 (11)
C91—C9—H9	107.2	C93'—C94'—H94'	126.6
C91'—C9—H9	116.0	O3'—C94'—H94'	126.6
C7—C9—H9	107.2	C91'—O3'—C94'	106.3 (9)
			
C9—N1—C2—C21	−176.84 (14)	C6—C7—C9—C91'	53.2 (5)
C9—N1—C2—C3	56.7 (2)	N1—C2—C21—C22	137.4 (2)
N1—C2—C3—C8	−54.15 (19)	C3—C2—C21—C22	−97.4 (2)
C21—C2—C3—C8	−178.38 (14)	N1—C2—C21—O2	−42.4 (2)
N1—C2—C3—C10	−174.73 (15)	C3—C2—C21—O2	82.80 (19)
C21—C2—C3—C10	61.0 (2)	O2—C21—C22—C23	0.1 (2)
N1—C2—C3—C4	62.0 (2)	C2—C21—C22—C23	−179.75 (19)
C21—C2—C3—C4	−62.3 (2)	C21—C22—C23—C24	0.1 (3)
C8—C3—C4—C5	53.4 (2)	C22—C23—C24—O2	−0.2 (3)
C10—C3—C4—C5	173.44 (17)	C23—C24—O2—C21	0.3 (2)
C2—C3—C4—C5	−63.7 (2)	C22—C21—O2—C24	−0.2 (2)
C3—C4—C5—C6	−46.9 (2)	C2—C21—O2—C24	179.64 (16)
C4—C5—C6—C7	46.7 (2)	N1—C9—C91—C92	42.8 (16)
C5—C6—C7—C8	−52.7 (2)	C91'—C9—C91—C92	7 (11)
C5—C6—C7—C11	−172.77 (18)	C7—C9—C91—C92	−82.9 (14)
C5—C6—C7—C9	64.3 (2)	N1—C9—C91—O3	−138.8 (8)
C10—C3—C8—O1	−7.6 (3)	C91'—C9—C91—O3	−175 (13)
C4—C3—C8—O1	111.5 (2)	C7—C9—C91—O3	95.6 (10)
C2—C3—C8—O1	−126.8 (2)	O3—C91—C92—C93	−2.0 (13)
C10—C3—C8—C7	176.76 (16)	C9—C91—C92—C93	176.5 (14)
C4—C3—C8—C7	−64.1 (2)	C91—C92—C93—C94	1.9 (11)
C2—C3—C8—C7	57.6 (2)	C92—C93—C94—O3	−1.3 (10)
C11—C7—C8—O1	7.1 (3)	C92—C91—O3—C94	1.2 (12)
C6—C7—C8—O1	−111.9 (2)	C9—C91—O3—C94	−177.5 (8)
C9—C7—C8—O1	126.7 (2)	C93—C94—O3—C91	0.2 (10)
C11—C7—C8—C3	−177.27 (17)	N1—C9—C91'—C92'	37 (3)
C6—C7—C8—C3	63.8 (2)	C91—C9—C91'—C92'	−177 (15)
C9—C7—C8—C3	−57.7 (2)	C7—C9—C91'—C92'	−83 (3)
C2—N1—C9—C91	177.1 (5)	N1—C9—C91'—O3'	−146 (3)
C2—N1—C9—C91'	−176.8 (13)	C91—C9—C91'—O3'	0 (9)
C2—N1—C9—C7	−56.8 (2)	C7—C9—C91'—O3'	95 (3)
C8—C7—C9—N1	54.4 (2)	O3'—C91'—C92'—C93'	−1 (3)
C11—C7—C9—N1	175.29 (16)	C9—C91'—C92'—C93'	176 (3)
C6—C7—C9—N1	−61.5 (2)	C91'—C92'—C93'—C94'	3 (2)
C8—C7—C9—C91	179.8 (2)	C92'—C93'—C94'—O3'	−3 (3)
C11—C7—C9—C91	−59.3 (3)	C92'—C91'—O3'—C94'	0 (3)
C6—C7—C9—C91	63.9 (3)	C9—C91'—O3'—C94'	−178 (3)
C8—C7—C9—C91'	169.1 (5)	C93'—C94'—O3'—C91'	2 (3)
C11—C7—C9—C91'	−70.0 (5)		

### Hydrogen-bond geometry (Å, º)

**Table:**

*D*—H···*A*	*D*—H	H···*A*	*D*···*A*	*D*—H···*A*
N1—H1···O1^i^	0.89 (2)	2.84 (2)	3.647 (2)	152.0 (13)

Symmetry code: (i) *x*, *y*+1, *z*.
